# Quantitative and qualitative aspects of standing-up behavior and the prevalence of osteochondrosis in Warmblood foals on different farms: could there be a link?

**DOI:** 10.1186/s12917-017-1241-y

**Published:** 2017-11-09

**Authors:** E. M. van Grevenhof, A. R. D. Gezelle Meerburg, M. C. van Dierendonck, A. J. M. van den Belt, B. van Schaik, P. Meeus, W. Back

**Affiliations:** 1 Animal Breeding and Genomics Centre, Wageningen UR Livestock Research, Wageningen, The Netherlands; 2 Department of Equine Sciences, Utrecht University, Utrecht, The Netherlands; 3 Behavioral Biology Group, Utrecht University, Utrecht, The Netherlands; 4 Department of Companion Animal Sciences, Division of Diagnostic Imaging, Utrecht University, Utrecht, The Netherlands; 5 GD-Animal Health Service, Deventer, The Netherlands; 6 Ridderkerk Equine Clinic, Ridderkerk, The Netherlands; 7 Department of Surgery and Anaesthesiology of Domestic Animals, Ghent University, Merelbeke, Belgium

**Keywords:** Joint level, Osteochondrosis, Sliding, Standing-up behavior, Warmblood foals, Horses

## Abstract

**Background:**

Osteochondrosis (OC) is a common, clinically important joint disorder in which endochondral ossification is focally disturbed. Reduced blood supply to growing cartilage is considered an important cause of the condition, which has both genetic and environmental origins. Housing conditions can influence cartilage injury through peak-pressure changes during limb sliding. Additionally, circulatory perturbation can cause the avascular necrosis of cartilage. In this study, we evaluated the type and frequency of limb sliding during standing up and the occurrence of OC in foals aged up to 12 months on different farms.

**Methods:**

Standing-up behavior was observed in 50 weaned, group-housed, Dutch Warmblood foals aged 6–9 months at five farms using black-and-white surveillance cameras, and their standing-up behavior was scored using a predetermined ethogram. OC was scored using a categorical scale between 6 and 12 months of age in 50 foals in the weanling period, and in 48 from the weanling to yearling periods because two foals died in this time.

**Results:**

At both 6 and 12 months of age, the total prevalence of OC differed between the farms: the lowest prevalence was observed on a farm with no sliding, and the highest prevalence was evident on a farm with a higher sliding frequency. The mean ratio of sliding versus normal standing-up behavior was 29% (range: 0–50%); i.e., foals experienced limb sliding during around 29% of standing-up maneuvres. The frequency of sliding instead of normal standing-up behavior differed significantly between the farms (range: 0–50%; *P* < 0.05), but significantly decreased when foals could better prepare themselves to stand, e.g., when there was an obvious provocation such as the announced approach of another foal (*P* < 0.05).

**Conclusions:**

Small but significant differences exist between farms in the sliding frequency and total OC incidence in Warmblood foals, but whether environmental factors are causally related to these differences requires further elucidation.

**Electronic supplementary material:**

The online version of this article (10.1186/s12917-017-1241-y) contains supplementary material, which is available to authorized users.

## Background

Osteochondrosis (OC) is a clinically important joint disorder that occurs in many animal species, including dogs, horses, pigs, and humans. It is defined as a focal disturbance of endochondral ossification and is believed to be a multifactorial disease. The most frequently described etiologic factors are heredity, rapid growth, trauma, and dietary imbalances, although its etiology remains incompletely understood [1–3]. Recently, it was shown in foals [4], pigs [5] and horses [6] that biomechanical damage to the juvenile blood supply plays an important role in the pathogenesis of OC [7]. Consistently, mRNA expression was demonstrated to differ in bone and cartilage after experimental mechanical loading between OC-negative and -positive foals [8]. Early OC lesions in pigs and foals appear to be very similar. In foals, OC is associated with the necrosis of cartilage vessels at between 3 weeks and 5 months of age. Cartilage canals are absent in foals aged over 7 months and, subsequently, nutrition is supplied to the cartilage through diffusion [4]. A recent study [9] showed that vessel failure is associated with ischemic necrosis in growing cartilage. However, OC is a dynamic disorder: during the first few months of life, lesions occur and repair spontaneously [10, 11]. This implies a “window of susceptibility” in which OC development and cartilage repair vary between joints [12, 13]. Dik et al. [10] showed that OC lesions become permanent from approximately 5 months of age in the tarsocrural (TC) joint, although disease stability is reached at a later stage; similarly, OC lesions become permanent from the age of approximately 8 months in the femoropatellar (FP) joint.

Exercise is an important environmental factor for musculoskeletal development in foals [1, 14], but is thought to have only an indirect effect on OC development [13]. Permanent box rest combined with exercise bouts every other day from 1 month of age did not significantly influence the number of OC lesions, but after 5 months of age, there was a tendency towards other, severe degenerative musculoskeletal lesions [13]. Recently, Van Grevenhof et al. [15] showed that rapidly growing pigs housed on conventional, slippery flooring developed OC significantly more frequently than those housed on a deep litter floor with restricted feeding. On standing up, one or more limbs can slip sideways as a result of weaker, less effective muscle function, a slippery floor, or a combination of these factors. Aberrant standing-up behavior may cause excessive changes in peak pressure on joint surfaces, damaging the vasculature of juvenile cartilage and resulting in avascular necrosis. Indeed, lateral wedge-induced, out-of-plane hindlimb movement has been shown to cause considerable asymmetrical loading of the fetlock, hock, and stifle joints in ponies [16, 17].

Acute joint overloading resulting from incidental, out-of-plane limb movements may cause the fragmentation of OC-prone cartilage. Lepeule et al. [18] reported that increased growth and reduced or irregular physical activity during the first weeks of life are responsible for lesions being more severe, whereas Praud et al. [19] found that “mixed housing” during winter and pastures with rough ground were significantly associated with OC with a poor radiologic score.

Based upon these epidemiologic findings, we hypothesized that farms in which foals experienced a higher sliding frequency, resulting in a greater number of incidental, out-of-plane limb movements, would have a higher prevalence of OC.

Therefore, in this study, we examined three questions:

1. Is there a difference between the standing-up behavior of foals between different farms during the first 12 months of life?
2. Is there a difference in OC development in foals between different farms during the first 12 months of life?
3. Is standing-up behavior linked to OC development in these foals during the first 12 months of life?

## Methods

### Foals

Five farms in The Netherlands were selected to participate in this pilot study, from which a total of 50 Dutch Warmblood foals were chosen for their immediate availability. Two foals died before the completion of the observations, so the data reported relate to 48 foals. The participating farms differed with respect to management factors, including their housing and exercise systems, and were ranked according to their sliding ratio. On average, 10 foals (range: 6–18 foals) per farm participated in the study. These foals were aged 6–12 months and their height at the withers ranged from 1.36–1.44 m.

### Observations of standing-up behavior

**Fig. 1:**
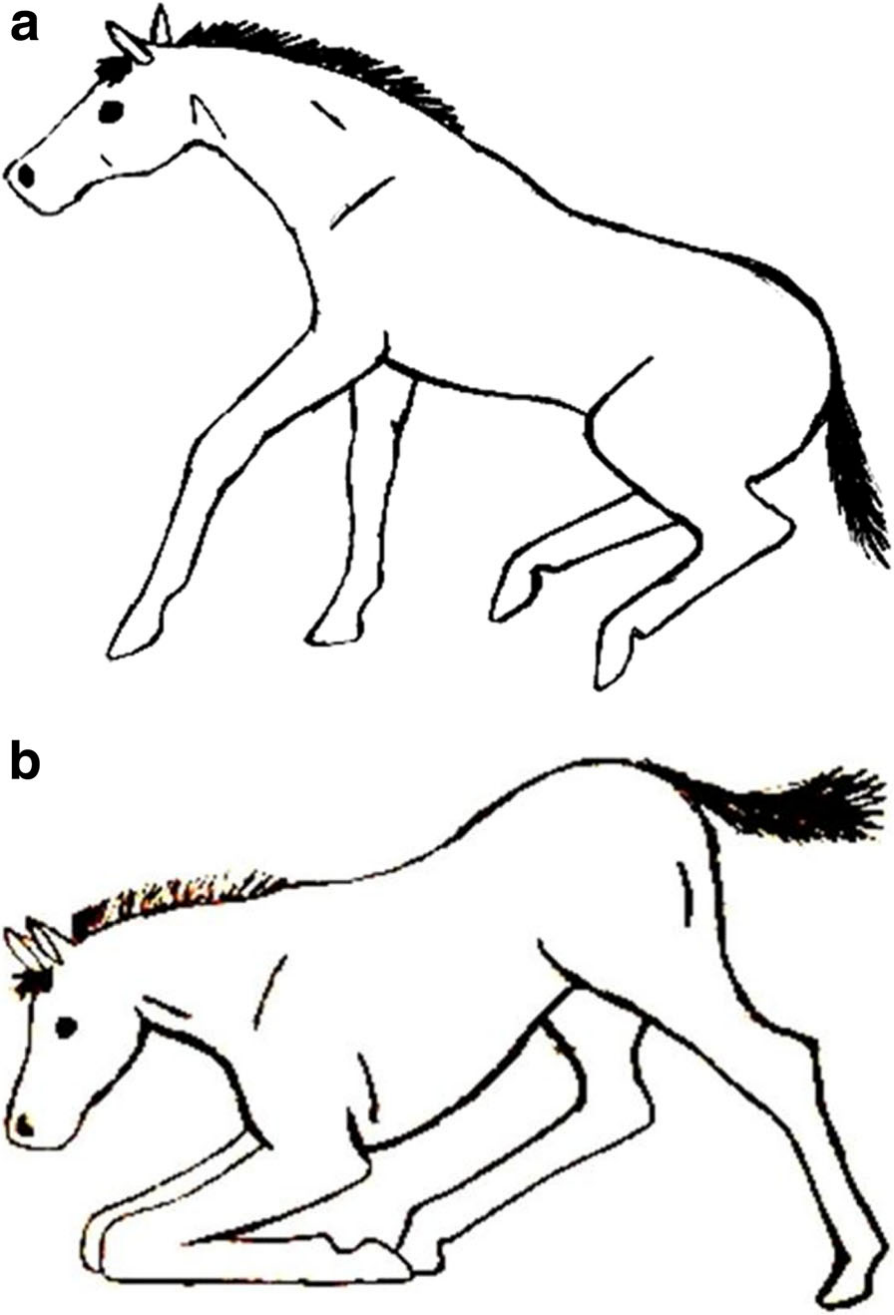
Schematic illustration of the way in which foals tend to stand up, as scored in this study: **a** the more common “forelimb-first” (equine) method, and **b** the less common “hindlimb-first” (bovine) method

Standing-up behavior was observed in weaned, group-housed foals aged 6–12 months at each farm; the sliding ratio (%) was calculated from the recorded number of incidences of sliding of the total number of recorded incidences of standing up. Standard black-and-white surveillance cameras (Mitsubishi) and video recorders (Sanyo) were used to record each group of foals for 3–4 h per session up to a total of 24 h per group, and the images were stored on videotapes. A video splitter device (EQM 100 Monoquad) was used to create one image containing different views of the stable. Each foal was scored using a predefined ethogram (Additional file 1) containing definitions of all the behaviors observed. Using the all-occurrences method [20], each standing-up maneuvre (when fully visible) was evaluated. Several characteristics were scored: baseline behavior (rolling; hindlimbs lying laterally left or right; or forelimbs lying sternally left or right); provocation (following another foal; external disturbance; panic; another cause; or cause not visible); standing-up behavior (forelimb or hindlimb first [Fig. 1]; direction of movement in relation to the center of gravity; normal or sliding [and which limb was sliding]; and the extent of sliding [short, medium, or long]).

### Scoring osteochondrosis

Standardized radiologic examinations (fetlock: LM, hock: AP/LM/APLO, and stifle: LM) were routinely performed at 6 and 12 months of age using standard mobile digital equipment. OC was scored by a board-certified radiologist from A–E using a categorical scale [10] as follows: A, no abnormalities; B, smooth flattening of the bony contour delineating the cartilage; C, slight irregular flattening of the bony contour delineating the cartilage; D, minor fragmentation; and E, a severely irregular ossification front with pronounced fragmentation. The OC ratio (%) was calculated from the recorded number of OC-positive joints of the total number of evaluated joints. Four joints were scored bilaterally for OC: the metacarpo- and metatarsophalangeal (fore and hind “fetlock”), TC (“hock”), and FP (“stifle”) joints. The original scores were subsequently converted to scores of 0–4 on a quantitative scale to emphasize the quantitative continuous characteristics of the trait, enabling summation at an animal level (quantitatively explained as follows: 1, a regular subchondral ossification front [SOF]; 2, focal smooth flattening of the SOF; 3, irregular flattening of the SOF; 4, minor [and what that was, such as fragments of 1–2 mm or <3 mm] fragmentation of the SOF; and 5, an irregular SOF with osteochondral fragments >3 mm). The degree or severity of OC at the animal level was obtained by the summation of the scores for all eight scored joints. For example, eight joints with the scores “0, 0, 3, 0, 1, 1, 0, 0”, would score “5” at the animal level. Only 39 radiographs at both 6 and 12 months were available for follow-up.

### Parameter estimation

In the univariate general linear models (Models 1 and 3, presented below), analyses and assessments of correlation (Pearson correlation coefficient) were performed using PROC GLM in SAS 9.2 (SAS Institute Inc., Cary, NC, USA; [21]). In the bivariate model (Model 2), parameter estimations and correlations were performed via the residual maximum-likelihood method using ASReml [22].

For the estimation of parameters, the following linear univariate general linear model was used:

1 $$ {\mathrm{Y}}_{\mathrm{i}\mathrm{jkl}}=\upmu +{\mathrm{farm}}_{\mathrm{i}}+\mathrm{OC}{12}_{\mathrm{j}}+{\mathrm{reason}}_{\mathrm{k}}+{\mathrm{e}}_{\mathrm{i}\mathrm{jkl}} $$

in which Y_ijkl_ is the frequency ratio of sliding versus normal standing-up behavior of an individual; μ is the mean frequency ratio; farm_i_ is the fixed-class effect of farm (*i* = 1, 2, …, 5); OC12_j_ is the continuous effect of OC at the animal level at 12 months of age (j = 0, 1, 2, …, 8); reason_k_ is the fixed-class effect of the reason for standing up, if any (k = 0, 1); and e_ijkl_ is the residual. All fixed effects in the model had a significance level of *P* < 0.05.

Additionally, the following bivariate model was used, in which we assumed the two processes of OC at the animal level and standing-up behavior were co-occurring:

2 $$ {\mathrm{Y}}_{\mathrm{tr}1}\ {\mathrm{Y}}_{\mathrm{tr}2}={\upmu}_{\mathrm{tr}1}+{\upmu}_{\mathrm{tr}2}+{\mathrm{farm}}_{\mathrm{tr}1}+{\mathrm{reason}}_{\mathrm{tr}1}+{\mathrm{e}}_{\mathrm{tr}1}+{\mathrm{e}}_{\mathrm{tr}2} $$

in which Y_tr1_ is the frequency ratio of sliding versus normal standing-up behavior; Y_tr2_ is the prevalence of OC at the animal level at 12 months of age; μ_tr1_ is the mean of Y_tr1_; μ_tr2_ is the mean of Y_tr2_; farm_tr1_ is the fixed-class effect of farm (*i* = 1, …, 5) on Y_tr1_; reason_tr1_ is the fixed-class effect of the reason for standing, if any (k = 0, 1) on Y_tr1_; e_tr1_ is the residual of Y_tr1_; and e_tr2_ is the residual of Y_tr2_. All fixed effects in the model had a significance level of *P* < 0.05. No traits significantly influenced Y_tr2_ (OC at 12 months of age) in the bivariate model.

Standing-up behavior with respect to whether foals rose “forelimbs first” or “hind limbs first” (Fig. 1) and the effect of this on OC prevalence were also analyzed. The following univariate model was used:

3 $$ {\mathrm{Y}}_{\mathrm{i}\mathrm{jkl}}=\upmu +{\mathrm{hindlimbs}\  \mathrm{first}}_{\mathrm{i}}+{\mathrm{e}}_{\mathrm{i}\mathrm{jkl}} $$

in which Y_ijkl_ represents OC in the hock joint at 6 months of age; μ is the mean prevalence; hindlimbs first_i_ is the fixed-class effect of the number of standing-up maneuvres performed with the hindlimbs first (*i* = 0, 1, 2); and e_ijkl_ is the residual. All fixed effects in the model had a significance level of *P* < 0.05. No other traits affected Y in Model 3. In general, all traits were tested in the model. Those traits that did not significantly influence the model were not taken into account in the models described above.

## Results

The mean ratio of sliding versus normal standing-up behavior was 29% (range: 0–50%), i.e., foals experienced limb sliding during around 29% of standing-up maneuvres, based on a mean of 46 observations of standing up per foal. Per foal, on average, there was a particular reason for the foal to stand up 43% of the time; the reason was most often the approach of another foal. The frequency ratio of sliding versus normal standing-up behavior and that of an identifiable reason for standing (following another foal; external disturbance; panic; or another cause) versus no visible reason were not significantly correlated. The sliding ratio was significantly determined by farm (*r* ^2^ = 0.323, *P* = 0.0072, *n* = 5), and was also significantly different between farms (one-factor analysis of variance *F* = 4.455, *P* < 0.005).

**Fig. 2:**
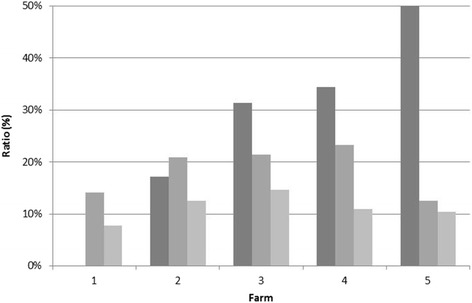
Bar chart displaying the sliding ratio (dark bar) and osteochondrosis ratio at 6 (medium-dark bar) and 12 (light bar) months of age for the farms listed on the *x*-axis in the order of their sliding ratio, with Farm 1 showing the lowest sliding ratio (0%) and Farm 5 the highest sliding ratio (50%)

The prevalence of OC at the animal level varied from zero to eight joints affected and 25% of foals were free of OC at 6 months of age (i.e., 75% OC). During the following 6 months, 23% of foals with OC recovered (i.e., 52% OC) and 10% developed OC (i.e., 62% OC), resulting in 38% of foals free of OC at 12 months of age (Fig. 2, Table 1). Around 52% of foals had OC at both 6 and 12 months of age. Table 1 presents the total OC scores. For example, when a foal scored 0 in the fetlock, 4 in the hock, and 2 in the stifle joint, the total OC score representing OC severity at the animal level was 6.

**Table 1:** The prevalence of osteochondrosis at the animal and joint levels among 48 foals aged 6 and 12 months

Variable	Age (months)	*n* (animals)	Mean OC score	SD	Minimum OC score	Maximum OC score
OC animal	6	40	2.7	2.7	0	12
OC hock	6	41	1.2	1.7	0	6
OC stifle	6	41	0.9	1.4	0	5
OC fetlock	6	40	0.6	1.3	0	6
OC animal	12	48	1.9	2.3	0	8
OC hock	12	48	0.9	1.7	0	8
OC stifle	12	48	0.4	0.9	0	4
OC fetlock	12	48	0.7	1.5	0	6

*n* number of animals available for each trait, *SD* standard deviation, *OC* osteochondrosis

In the stifle joint, the total OC severity varied from 0 to 4 at 12 months of age (Table 1; warmblood studbook score: A-E), whereas the OC prevalence decreased from 38% at 6 months to 26% at 12 months of age. In the hock joint, the total OC severity varied from 0 to 8 at 12 months of age (Table 1), whereas the OC prevalence decreased from 41% at 6 months to 28% at 12 months of age. In the fetlock joint, the total OC severity varied from 0 to 6 at 12 months of age (Table 1), whereas the OC prevalence decreased from 28% at 6 months to 18% at 12 months of age.

In general, the OC prevalence at 6 months showed a significant correlation with that at 12 months of age for all joints (*r* = 0.46, *P* = 0.003), but also for each individual joint, with *r* varying per joint from 0.6–0.67 (*P* < 0.01) at 6 months and 0.35–0.67 (*P* < 0.02) at 12 months of age. Moreover, individual joints showed a significant within-joint correlation at both ages in OC prevalence, with *r* varying from 0.41–0.43 (*P* < 0.01).

The univariate Model 1 demonstrated that “farm”, “foal OC score at 12 months of age”, and “reason to stand” were significantly and positively associated with “sliding ratio” (*r* ^2^ = +0.52, *P* < 0.05; Tables 2 and 3). The bivariate Model 2 demonstrated that “foal OC score at 12 months of age” and “sliding ratio” were significantly but negatively correlated (*r* ^2^ = −0.36, *P* < 0.05). Both “farm” (positively) and “reason to stand” (negatively) significantly influenced “sliding ratio” (*P* < 0.05). None of the variables significantly influenced OC at the animal level at 12 months of age. Finally, the univariate Model 3 examined the method of standing-up (whether foals rose using either their forelimbs or hindlimbs first [Fig. 1]) with respect to its effect on OC development. Use of the hindlimbs first (Fig. 1) showed only a slight tendency to be related to OC prevalence in the hock joint at 6 months of age (*r* ^2^ = 0.094, *P* = 0.057).

**Table 2:** Model parameters (univariate) affecting the group of 48 foals for the farms listed in order of sliding ratio, with Farm 1 showing the lowest sliding ratio (0%) and Farm 5 showing the highest sliding ratio (50%) The farms (*n* = 5) are listed 1–5 (detailed information provided in Table 3)

Parameter	Subcategory of OC	Estimate/LSM	*P*
Intercept		+0.07	<0.001*
Farm			<0.001*
	1	−0.16	0.099
	2	+0.38	<0.001*
	3	+0.27	0.003*
	4	+0.35	<0.001*
	5	+0.41	<0.001*
OC at 12 months		−0.03	0.018*
Specific reason for standing-up versus no reason		−0.41	0.005*

Least squares means are presented at the farm level; standard deviation varied from 0.05–0.1 (*P < 0.05). The most frequent reason for standing up was the approach of another foal; OC = osteochondrosis; LSM = least squares mean

**Table 3:** Schematic tabular overview of the differences between Farms 1–5 listed in order of sliding ratio, with Farm 1 showing the lowest sliding ratio (0%) and Farm 5 showing the highest sliding ratio (50%)

Farms	1 (*n* = 8)	2 (*n* = 6)	3 (*n* = 12)	4 (*n* = 18)	5 (*n* = 6)
Average birth month	April	April	April	April	June
Average withers height	1.4 m	1.37 m	1.36 m	1.44 m	1.4 m
Stable size (avg/foal)	1250 m^2^	18.3 m^2^	8 m^2^	5 m^2^	7.5 m^2^
Cleaning frequency	1/wk	1/wk	1/wk	1/wk	2–3/wk
Feeding amount	2 kg/d	1 kg/d	2 kg/d	n.a.	2–4 kg/d
Roughage	Silage	Silage	Hay	Silage	Silage
Ground flooring	Bricks/sand	Straw bed	Straw bed	Straw bed	Straw bed
Exercise area	Pasture	Arena	Paddock	Paddock	Arena
Exercise duration/d	24 h/d	0.5–1 h/d	1–2 h/d	1–1.5 h/d	3–4 h/d
Hoof trimming	No	Yes	Yes	Yes	Yes

*n.a.* not available, *d* day, *wk.* week, *avg.* average

## Discussion

We hypothesized that farms in which foals showed a higher sliding frequency would house animals that experienced more incidental, out-of-plane limb movements. We also speculated that farms with a higher sliding frequency would exhibit a higher prevalence of OC. Each farm used a different type of housing and this was associated with the ratio of sliding versus normal standing-up behavior shown by the foals. The management variations between the horse farms evaluated in this study were similar to those found in pig farming, in which both deep litter and conventional flooring systems are used: the frequency of renewal ranged from 1 to 3/wk., and the type of bedding provided ranged from straw to no bedding at all. The exercise area and duration/day differed between the farms (Table 3): Farm 5 gave the longest “active” exercise duration to their foals (3–4 h/day) in contrast to the longer “passive” pasture exercise given at Farm 1. Generally, moderate exercise improves the circulation and development of cartilage and bone, thereby influencing the risk of OC development [13]. Differences in management at individual farms were not identified as causal factors for sliding behavior by our statistical analyses, but only five farms participated in this study.

The prevalence of OC changed significantly over time at the animal level, and also within each joint (*P* < 0.05). The process of OC development and recovery varied over time and differed depending on the joint affected [23]. Both the development of new lesions and fragments and the repair of OC lesions were observed between the ages of 6 and 12 months, confirming early reports [13]. OC was found most frequently and most severely (scores D and E) in the hock joints (not shown), followed by the stifle joints, and finally the fetlock joints.

The prevalence of OC at the animal level at 12 months of age was somewhat negatively correlated with the sliding ratio; possibly, pain may have contributed to some animals showing sliding during standing up less frequently. This implies that, in foals suffering from OC, the resulting pain caused relatively careful standing-up behavior, as significantly less sliding behavior was evident.

The development of OC in joints before 6 months of age depends on many different factors, of which standing-up behavior is just one (Fig. 2, Table 3). After 6 months, it remains possible for OC to develop and repair. The process of repair is also multifactorial and the time frame (window of susceptibility) differs between joints [23]. A relatively high, significant correlation was found between the prevalences of OC at 6 and 12 months of age (Pearson’s *r* = 0.36, *P* < 0.05), but the OC prevalence at 6 months (19.1%) was significantly higher than that at 12 months (10%; paired *t* test; *P* < 0.05), in accordance with earlier studies on OC prevalence [13].

Only the number of times a foal stood up with its hindlimbs first showed a tendency (*P* = 0.057) to be related to OC in the hock joint at 6 months of age. From a practical perspective, the method of standing up can be expected to have an effect on OC development in foals. Lifting the body by raising the hindlimbs first is usually seen in calves, in contrast to the forelimb-first approach more commonly used by foals. A tendency towards a correlation between sliding during standing up and OC was evident in the hock joint alone and only at 6 months of age. The association with OC in the hock joint at 12 months was not significant. In this respect, we assume that foals who often stand up hindlimbs first aged 6–12 months will also have done so aged 0–6 months, and anticipate a stronger statistical effect with a larger sample size. It can also be expected that the differences in OC prevalence between the housing systems and specific management factors evaluated would become significant were more farms to participate in this type of study.

## Conclusions

The ratio of sliding versus normal standing-up behavior differed significantly between farms and depended on the reason for standing up, such as the approach of another foal. The prevalence of OC at the animal level at 12 months of age was negatively correlated with the ratio of sliding during standing up, but it is possible that pain might have prevented the animals from showing frequent sliding. There are small but significant differences between farms in sliding frequency and the total incidence of OC in their Warmblood foals, but whether environmental factors are causally related to these differences requires further elucidation.

## Additional file

Additional file 1: Ethogram standing-up behaviour in foals. (DOCX 24 kb)

## Abbreviations

AP: Anterior-posterior
APMO: Anterior-posterior, medial oblique
FP: Femoropatellar
LM: Lateromedial
OC: Osteochondrosis
SOF: Subchondral ossification front
TC: Tarsocrural
