# Supplementary material for: Quantitative and qualitative aspects of standing-up behavior and the prevalence of osteochondrosis in Warmblood foals on different farms: could there be a link?
Source: BMC Vet Res. 2017 Nov 9;13:324. doi: 10.1186/s12917-017-1241-y (PMC5679338; doi:10.1186/s12917-017-1241-y)
Supplement: Additional file 1: — Ethogram standing-up behaviour in foals. (DOCX 24 kb) [file 12917_2017_1241_MOESM1_ESM.docx]

**Additional file 1**

**Ethogram for standing-up behaviour in foals**

**General information**
Farm:
Group:
Bedding:
Stable size:
Number of foals per stable:

Name of foal:
Sex:
Date of Birth:
Pedigree:
Markings:
Time of observation:
Location of observation:
Observer:
Detailed findings:

Observation was performed following the all-occurrences method (with an ethogram concentrating on details of the standing-up behavior and its provocation). This means that only the pre-described actions and determinants were observed for each animal during the observation period. All behaviours over the predetermined time were noted per foal; in this study, we observed only standing-up behavior.

1. Baseline behavior

|  | **Description** | **Code** | **#** |
| --- | --- | --- | --- |
| **Rolling** | Foal moves from one side to another, limbs are moving in the air. | ROL |  |
| **Lying laterally left** | Foal lies lateral, limbs to the left side. | LLL |  |
| **Lying laterally right** | Foal lies lateral, limbs to the right side. | LLR |  |
| **Lying sternally left** | Foal lies sternal, hindlimbs to the left side. | LSL |  |
| **Lying sternally right** | Foal lies sternal, hindlimbs to the right side. | LSR |  |

|  | **Description** | **Code** | **#** |
| --- | --- | --- | --- |
| **Not visible** | No indication visible inside the observation area that causes standing-up behavior. | ANZ |  |
| **Following another foal** | Another foal triggers standing-up behavior by the observed foal. | AAV |  |
| **External disturbance** | An external disturbance triggers the observed foal to stand up, for example a person. | AVR |  |
| **Panic** | Panic reaction within the group; all foals are frightened. | APG |  |
| **Other** |  | AAA |  |

1. Provocation
2. Standing behavior

|  | **Description** | **Code** | **#** |
| --- | --- | --- | --- |
| **Forelimbs first** | Stretching forelimbs to the front. | OVV |  |
| **Hindlimbs first** | Hindquarters raised first while forelimbs were folded under the body. | OAO |  |
| **Direction of movement in relation to the center of gravity** | Pushes weight to the other side during standing up. | OVG |  |
| **To the front** |  | VGV |  |
| **To the back** |  | VGA |  |
| **Sliding during standing up** | One limb slipped outside the center of gravity. | OGM |  |
| **Forelimb, left** |  | GLV |  |
| **Forelimb, right** |  | GRV |  |
| **Hindlimb, left** |  | GLA |  |
| **Hindlimb, right** |  | GRA |  |
| **Extend of sliding** |  | GMU |  |
| **Short** | 0–20 cm. | MUK |  |
| **Medium** | 20–50 cm. | MUM |  |
| **Long** | 50–100 cm. | MUV |  |
| **Standing foal** |  |  |  |
| **Firm** | Foal is standing firmly on its limbs without any staggering. | OVS |  |
| **Staggering** | Foal is standing weakly on its limbs and may slide or fall at any minute. | OVW |  |

1. Hindlimb slides

|  | **Description** | **Code** | **#** |
| --- | --- | --- | --- |
| **Bent stifle and tarsus, expressed in degrees** | Bent stifle and tarsus. | KTG |  |
| **90º** |  | NGG |  |
| **45º** |  | VGG |  |
| **Direction of movement to the back quarters during sliding** |  | GAG |  |

1. Reserve codes

|  | **Description** | **Code** | **#** |
| --- | --- | --- | --- |
| **Foal jumps** | No standing-up behavior observed because of a fright reaction by the foal. | VSO |  |
| **Foal plays and slides** | During playing around, the foal slides with one or more limbs. | SGO |  |
| **Other unexpected behavior** |  | AOB |  |
